# Sleep quality in Spanish university professors: Association with lifestyle habits and physical and mental health indicators

**DOI:** 10.1371/journal.pone.0320352

**Published:** 2025-04-07

**Authors:** Maria Estela Colado Tello, Josep María Dalmau Torres, Esther Gargallo Ibort, Raúl Jiménez Boraita

**Affiliations:** 1 University of La Rioja, Logroño, Spain; 2 Universidad Internacional de La Rioja, Logroño, Spain; Hamasaki Clinic, JAPAN

## Abstract

**Background:**

There is currently a growing interest in knowing the state of health of university professors. Sleep habits are essential for health and performance in any profession, including university teaching, and are related to different aspects of a teacher’s work productivity and general wellbeing.

**Objective:**

The study aimed to analyse sleep quality in Spanish university professors and to determine its association with lifestyle habits and physical and mental health indicators.

**Method:**

The study was carried out on a sample of 1560 university professors (47.39 ± 11.29 years) from thirteen universities belonging to the Spanish Network of Health Promoting Universities. Sleep quality, emotional exhaustion, depersonalisation, self-fulfilment, quality of life, stress, anxiety, depression, vocal fatigue, sedentary time and eating behaviour were assessed.

**Results:**

Thirty-three percent of university professors were found to have poor sleep quality (95% CI: 30.7% - 35.3%). Significant differences were identified based on sociodemographic factors, particularly gender, with 74.9% of men reporting good sleep quality compared to 50.5% of women. According to the regression analysis, sleep problems were associated with less improvement in vocal symptoms after rest and lower quality of life scores. Additionally, they were linked to greater vocal fatigue, physical discomfort in the voice, female gender, and higher scores in mental health disorders (stress, anxiety, and depression). University professors with poor sleep quality also showed lower levels of physical activity, increased sedentary time, and higher levels of uncontrolled and emotional eating.

**Conclusions:**

Enhancing sleep quality and rest among university professors can boost cognitive and physical performance, foster better overall well-being, and lead to higher job satisfaction.

## Introduction

There is currently a growing interest in understanding the state of health of university professors, analysing various occupational, personal and social elements that can influence both as triggers and disruptors of their wellbeing and quality of life, which could have a direct impact on academic performance and general wellbeing [1,2,3].

The different changes and demands of the education system require a high level of responsibility, involvement and attention from university professors, which can have negative consequences on their general health [4,5]. Teaching goes beyond the simple act of transmitting knowledge in a given subject [6,7], requiring a multidisciplinary dedication that exposes teachers to different health risks derived from their professional practice [8,9]. The academic load to which they are often subjected often exceeds their physical and mental capacities [10,11], which can affect both the quality of teaching and their active contribution in the educational institution [5,12].

The literature points to a direct relationship between lifestyle habits and teaching, as these can have a direct impact on their professional performance and the quality of education they provide [13,14]. As such, sleep habits are essential for health and performance in any profession, including university teaching, and are related to different aspects of a teacher’s work productivity and general wellbeing [15,16]. Sleep is defined as a reversible state of reduced awareness and responsiveness to stimuli [17]. This physiological activity is fundamental to life and is associated with numerous problems, both physical and psychological [18,19]. The American Academy of Sleep Medicine (2015), together with the Sleep Research Society, recommend an average of seven or more hours per day for adults aged 18-64 years to promote a healthy state of quality sleep [18,20]. However, several studies establish a prevalence of sleep disorders in adults ranging from 37% to 70%, being more pronounced in women and the elderly [21,22].

Societal evolution and transformation have led to an increase in the use of communication and information technologies, which has led to a trend towards a reduction in both sleep time and sleep quality [7,23]. A study by Muñoz-Pareja [22] revealed a worldwide prevalence of 35-45% of the adult population with sleep disorders. Furthermore, when focusing on the teaching population, although research is limited and mostly focused on pre-university stages, several studies indicate that between 30%-60% of teachers experience problems in sleep quality [18,19].Scientific evidence warns of the significant public health impact of poor quality and deficient sleep, constituting a relevant risk factor in the development of physical, psycho-emotional and social disorders and diseases [18,23,24]. Various research studies have highlighted how important university professors’ state of health is and its link with their commitment to academic performance, identifying stress and burnout as risk factors that, among others, lead to a deterioration in the quantity, quality and characteristics of sleep [7,25]. Accordingly, a high prevalence is observed in the education sector, especially in higher education, with an estimated 40% of university professors being affected [5,26,27].

In addition, sleep plays a crucial role in the body’s dynamic balance, and its disruption increases the risk of experiencing physical health-related problems, such as decreased immune competence or impaired and unstable physical performance [19,24]. Specifically, studies of university professors have found a clear relationship between sleep quality and cardiometabolic risk factors, such as hyperglycaemia, low HDL cholesterol and obesity, which is more pronounced in older men [7,18].

Along the same lines, there is evidence linking sleep to the lifestyles adopted. The scientific literature indicates that poor sleep quality is associated with a higher propensity to adopt unhealthy eating habits, which translates into an increased intake of fast food or caloric food at night, consumption of soft drinks and a lower motivation to engage in physical activity [18,21,28].

On the other hand, a subjective perception of poor sleep quality has been documented to be directly related to Burnout Syndrome, difficulties in emotional regulation, cognitive impairment and stress in work relationships [18,19]. Thus, current work requirements in university teaching may lead to a reduction in sleep hours and a decrease in sleep quality, which could manifest itself in a deterioration in burnout, depression, and work-related stress [7,18,29]. Furthermore, this exhaustion and emotional disturbances, along with stress and anxiety, are reported to be the result of inadequate non-restorative sleep, which impacts on teachers’ overall wellbeing and, by extension, their professional performance [15,19,24].

## Materials and methods

### Study design and participants

The study was conducted using a descriptive-correlational cross-sectional methodology and was carried out through an online survey. The sample was selected by means of convenience sampling, with the study population being university professors belonging to the Spanish Network of Health Promoting Universities. The final sample consisted of 1560 university teachers from thirteen universities, 779 men (49.9%) and 781 women (50.1%), aged between 23 and 74 years (M =  47.39, SD =  11.29).

### Procedure

The ethical foundations of the Declaration of Helsinki were respected at all times during the research process, and prior approval of the study was obtained from the Research Ethics Committee of the University of La Rioja. Participants were invited to complete the survey via email, where information about the purpose of the study was provided and online informed consent for participation was requested prior to accessing the questionnaire. The collaboration of the research participants was voluntary and anonymous. The questionnaire was sent to professors via the teaching email of the participating universities, introducing the study and providing access to the survey via a link to the SurveyMonkey platform. Potential participants had 90 days to respond, during which time two reminders with the link to participate were sent to those who had not yet responded. The data collection period took place between November 2023 and January 2024.

### Instruments

Sleep quality was assessed using the Pittsburgh Sleep Quality Index (PSQI), developed by Buysse [30] and validated and adapted to the Spanish case by Royuela and Macías [31]. The instrument assesses sleep quality and its clinical disturbances during the last month through nineteen items grouped into seven different sleep components: sleep quality, sleep latency, sleep duration, sleep efficiency, sleep disturbances, use of sleep medication and daytime dysfunction. Each component scores between zero and three after the calculation procedure of the dimensions defined by the instrument author. The sum of the seven components gives the total PSQI score, which ranges from 0 to 21. Higher scores indicate poorer sleep quality. The results were categorized into two groups based on sleep quality: ‘Good-quality sleep’ (score ≤  5) and ‘Poor-quality sleep’ (score >  5).

To estimate physical activity, the International Physical Activity Questionnaire Short Form (IPAQ-SF), validated in 12 countries, including Spain by Craig [32]. The IPAQ-SF estimates the intensity, frequency and duration of PA performed in the last seven days. This information was obtained through 7 questions related to the number of days of physical activity (intense, moderate and walking) and the daily time spent doing physical activity. The total weekly amount of PA was calculated according to the method of calculating the metabolic equivalent of the task (MET) minutes/week, previously set out in the IPAQ instructions for questionnaire data analysis and processing (IPAQ, 2005). The measures obtained from the questionnaire were, firstly, total physical activity expressed in MET-minutes.

Time spent on sedentary behaviours was estimated using the short version of the Sedentary Behavior Questionnaire (SBQ-S), adapted and validated in the Spanish population by Munguia-Izquierdo et al. [33]. The questionnaire consists of eleven items assessing the time spent on sedentary behaviour during the week and at the weekend (watching TV, listening to music, etc.). There are nine response options (“none”, “15 minutes or less”, “30 minutes”, “one hour”, “two hours”, “three hours”, “four hours”, “five hours”, “six hours or more”). The calculation of weekly sedentary time is calculated through a sum of all items and can be broken down into weekday and weekend sedentary time.

To assess eating habits, the Tree Factor Eating Questionnaire-R18 (Tfeq-Sp), a shortened version of the original TFEQ by Stunkard and Messick [34] and adapted and validated in the Spanish population by Jáuregui-Lobera [35]. The questionnaire measures three dimensions of eating behaviour: (a) cognitive restraint (6 items; conscious restriction of food intake); (b) uncontrolled eating (9 items; tendency to eat more than usual as a consequence of a loss of hunger control); and (c) emotional eating (3 items; eating in response to different negative emotions). The questionnaire consists of 18 items on a 4-point response scale (definitely true, mostly true, mostly false, definitely false). Scores are obtained by summing the items in each of the dimensions, with higher values representing more restricted, uncontrolled and emotional eating.

Health-related quality of life was assessed using the World Health Organization Quality of Life Scale (WHOQOL), in its abbreviated version WHOQOL-BREF developed by the World Health Organization [36]. The questionnaire assesses the perception of one’s own health status, psychosocial status and other aspects of quality of life during the last two weeks, through 26 5-point Likert scale items which in turn are grouped into four dimensions: physical health (7 items), psychological health (6 items), social relations (3 items) and environmental health (8 items). In addition, the questionnaire adds two items at the beginning of the questionnaire that assess the general perception of the participants’ quality of life and health. The score for each domain is used to calculate a raw score for that domain. Following the guidelines provided by the WHO, the sum of the different raw scores can be converted to a scale from 0 to 100 points, where higher scores indicate a higher quality of life. Additionally, an overall HRQoL score was calculated by summing the scores of the four domains and then dividing them by four.

Mental disorders were assessed using the Depression, Anxiety and Stress Scale (DASS-21), developed by Lovibond and Lovibond [37] and validated in the Spanish population by Fonseca-Pedrero [38]. This questionnaire analyses the negative emotional state experienced in the last week through 21 4-point Likert scale items: from 0 (has not happened to me) to 3 (has happened to me a lot, or most of the time). The instrument also consists of three 7-item subscales assessing depression, anxiety and stress. The score for each subscale was calculated by adding the items corresponding to each subscale. The sum of the scores obtained in each subscale was multiplied by 2 to make the results of the DASS-21 and DASS-42 comparable, so that the score for each subscale could be between 0 and 42. Higher values are associated with higher rates of stress, anxiety and depression. Additionally, the total score of the questionnaire was calculated by summing the values of the three dimensions.

The Maslach Burnout Inventory Educators Survey (MBI-ES), designed by Maslach and Jackson (1981) and adapted for Spanish educators [39], was used to assess Burnout Syndrome. This instrument assesses job burnout in the teaching profession through 22 items on a seven-point Likert scale: from 0 (never) to 6 (every day). The instrument consists of three independent subscales (emotional exhaustion, depersonalisation and self-fulfilment), where the score for each of them was obtained by adding the scores of their items. High scores on the first two subscales and low scores on the third define burnout syndrome.

Finally, the Vocal Fatigue Index (VFI) questionnaire developed by Nanjundeswaran [40] was used analysis of vocal fatigue and validated in Spanish university professors by Contreras-Regatero [41]. The questionnaire consists of 19 items related to possible symptoms associated with vocal fatigue due to voice use, which are grouped into three factors: “vocal fatigue and avoidance of voice use” (Factor 1; 11 items), “physical discomfort (Factor 2; 5 items) and “improvement of symptoms with rest” (Factor 3; 3 items). The items have five response options: never (0), hardly ever (1), sometimes (2), almost always (3) and always (4). The scores are obtained by adding the scores of the items in each dimension. High values in factors 1 and 2 are indicators of increased severity of vocal fatigue, while high values in factor 3 are indicators of improvement of vocal fatigue symptoms.

### Statistical analysis

Quantitative variables are represented according to their means and standard deviations, while qualitative variables are represented according to their frequencies. The normality and homoscedasticity of the data were studied with the Kolmogorov-Smirnov test and Levene’s test, respectively. The contrast of means was performed with the ANOVA test for variables with normal distribution and the Kruskal-Wallis H test for those with non-normal distribution. Pearson’s chi-square test was used to analyse the association between qualitative variables.

Multiple linear regression modelling using the backward elimination method was developed to identify variables associated with sleep quality. The variables included in the model were: physical wellbeing, mental wellbeing, environmental wellbeing and social relationships, stress, anxiety and depression, vocal fatigue, physical voice discomfort and improvement of voice symptoms with rest, emotional exhaustion, depersonalisation and self-fulfilment, physical activity, sedentary time, restricted eating, binge eating, emotional eating. Statistical analysis was carried out using IBM-SPSS® version 25 for Windows. Statistical significance was set at p <  0.05.

## Results

The prevalence of poor sleep quality among university professors was 33% (95% CI: 30.67% - 35.33%). Significant differences were identified based on sociodemographic factors, particularly gender, with 74.9% of men reporting good sleep quality compared to 50.5% of women. In contrast, no significant differences were observed in relation to age (Table 1).

**Table 1:** Sleep quality as a function of gender and age.

		N	Good-quality sleep	Poor-quality sleep	P Value
			%	%	
Gender	Man	776	74.9	25.1	<0.001
	Woman	784	59.4	40.6	
Age	20-30	107	71.30	28.70	0.447
	31-40	201	64,8	35.2	
	41-50	270	64.1	35.9	
	51-60	327	67.3	32.7	
	** ≥** 61	140	72.5	27.5	

Quality of life, burnout, mental disorders and vocal fatigue as a function of the level of sleep quality are presented in Table 2. The analysis found that university professors with poor sleep quality have higher levels of stress, anxiety, depression, emotional exhaustion, depersonalisation and voice fatigue (vocal fatigue and avoidance of voice use and physical voice discomfort). They also show less improvement of voice symptoms with rest, self-fulfilment and quality of life indices in all dimensions.

**Table 2:** Quality of life, stress, anxiety, depression and vocal fatigue as a function of sleep quality.

	Good-quality sleep N = 1045		Poor-quality sleep N = 515		P Value
	M	SD	M	SD	
Physical wellbeing	76.75	13.07	62.65	15.86	<0.001
Mental wellbeing	70.96	15.73	60.06	18.07	<0.001
Environmental wellbeing	71.35	14.60	65.50	15.77	<0.001
Social wellbeing	65.21	19.96	58.32	21.10	<0.001
Stress	7.76	7.73	14.36	9.75	<0.001
Anxiety	3.40	4.46	7.20	7.72	<0.001
Depression	4.28	6.43	8.93	9.52	<0.001
Vocal fatigue and avoidance of voice use	8.46	8.05	11.80	10.08	<0.001
Physical voice discomfort	2.40	3.39	3.79	4.45	<0.001
Improvement of voice symptoms with rest	5.31	4.17	4.17	3.18	<0.001
Emotional exhaustion	16.01	10.47	22.18	11.96	<0.001
Depersonalisation	2.57	3.32	3.19	3.75	0.001
Self-fulfilment	32.93	9.02	31.86	8.77	0.013

Table 3 Shows an analysis of physical activity, sedentary time and eating behaviour according to the dimensions of sleep quality. Lower levels of PA and more sedentary time were found in university professors with poor sleep quality. Furthermore, in relation to eating behaviour, university professors with poor sleep quality had higher levels of uncontrolled and emotional eating.

**Table 3:** Physical activity, sedentary time and eating behaviour according to the dimensions of sleep quality.

	Good-quality sleep N = 1045		Poor-quality sleep N = 515		P Value
	M	SD	M	SD	
METS	2752.98	2979.76	2351.22	2784.12	<0.001
Time sedentary	3490.64	1008.96	3740.05	1122.19	<0.001
Cognitive restraint	15.67	4.30	15.80	4.46	0.770
Uncontrolled eating	15.75	4.47	17.31	5.48	<0.001
Emotional eating	5.34	2.22	6.15	2.49	<0.001

Finally, Table 4 presents the multiple linear regression model related to sleep problems. These issues were associated with lower levels of improvement in voice symptoms with rest and WHOQOL-BREF scores, while being linked to higher levels of vocal fatigue and avoidance of voice use, physical voice discomfort, female gender, and DASS-21 scores.

**Table 4:** Regression analysis in relation to sleep quality.

	B	SD	Standard B	t	P Value	95% CI	R2
Vocal fatigue and avoidance of voice use	0.021	0.010	0.072	2.040	0.041	0.001- 0.041	0.255
Physical voice discomfort	0.078	0.023	0,116	3.417	0.001	0.033 - 0.123	
Improvement of voice symptoms with rest	-0.140	0.017	-0.213	-8.496	<0.001	-0.173 - -0.108	
Gender (female)	0.571	0.115	0.111	4.946	<0.001	0.345 – 0.798	
Dass-21	0.025	0.004	0.199	6.713	<0.001	0.018 – 0.033	
WHOQOL-BREF	-0.037	0.005	-0.205	-7.139	<0.001	-0.048 - -0.027	

## Discussion

This study’s results show the association between sleep quality and various lifestyle habits and physical and mental health indicators. The study shows that 33% of university professors have poor sleep quality. Although studies on university professors’ sleep quality-related state of health are scarce, these prevalences are similar to those obtained in other research in various countries such as Brazil and China, which showed high rates of poor sleep quality among university professors [19,21]. Accordingly, this study builds on previous research highlighting concerns about the high number of teachers with poor sleep quality, underlining that this profession is characterised by high demands and work overload [7,42]. It is reflected how factors such as gender, quality of life and symptoms related to vocal fatigue are of particular importance in relation to sleep quality in university teachers. Thus in our study, notable differences related to sociodemographic factors were found, especially in terms of gender, as 74.9% of men reported enjoying good sleep quality, compared to 50.5% of women. These findings are consistent with other research that also indicates a higher prevalence of sleep problems in women (44.4%) compared to men (41.5%), adding biological hormonal differences as a cause of this difference [43]. Other studies reflect how 36.2% of women experience daytime sleepiness compared to men (34.1%) [44]. This could have important implications for health and well-being, as daytime sleepiness can influence productivity, mood and overall quality of life [44,45].

Regarding health-related quality of life, university professors with good sleep quality were found to have higher levels in all four dimensions of quality of life analyzed (physical wellbeing, mental wellbeing, environmental wellbeing, and social relationships). This was confirmed in the regression analysis, where the total score of the WHOQOL-BREF questionnaire was negatively associated with sleep-related problems. These results coincide with those found in other studies that confirm positive associations between sleep disorders and quality of life in all its dimensions, highlighting the need for early detection, prevention and promotion [24,46]. Likewise, sleep and its alterations are considered an excellent indicator of general health and quality of life [17,47]. Although studies in relation to university professors are scarce, the educational environment is highlighted as a vulnerable and changing environment, which has an impact on the quality of sleep and, therefore, on the quality of life of university professors [24]. This research concludes that the fast pace inherent in the teaching profession, together with the additional workload it entails, has a particular impact on their rest and sleep, with overt daytime sleepiness, and consequently on their quality of life.

In terms of mental disorders, university professors with poor sleep quality showed higher levels of stress, anxiety, and depression. Additionally, the regression analysis confirmed a positive association between the total DASS-21 score and sleep problems. Research confirms that disturbances in sleep quality become the main driver of other physical and psycho-emotional illnesses, including stress, anxiety and depression [48,49]. Although studies with university professors are scarce, research such as that of Akçay [50] demonstrates a significant relationship between anxiety and sleep disorders in academic university professors and identifies anxiety as the most common psychiatric disorder in people with sleep disturbances. In turn, other studies corroborate the bidirectional relationship between sleep disorders and depression. Research by Alqurashi [51] with university professors and students concludes that poor sleep quality is associated with depressive symptomatology. As for stress, authors such as de Sousa [45] show a deficiency in the adaptive resources of teachers in the face of the work-related stress they experience in their day-to-day work as teachers. Moreover, they manifest a reality which leads to problems in the conciliation of sleep and states of insomnia, even to the need to consume hypnotic and anxiolytic pharmacology.

As for vocal fatigue, university professors with poor sleep quality showed higher levels of fatigue and physical voice discomfort, as well as lower rates of recovery from vocal fatigue symptoms. Additionally, the regression analysis confirmed these associations. These results are in line with previous studies indicating a higher prevalence of voice disorders and burnout among university professors with sleep disturbance or poor sleep quality [52]. Thus, Carrillo-Gonzalez [21] identified a higher frequency of vocal fatigue among professors who sleep less than 6 hours a day. Along the same lines, studies with teachers at stages other than university have shown that not only occupational factors are the cause of vocal fatigue, but that teachers’ lifestyle habits, such as diet and sleep, are also clearly important [53]. The literature indicates that current work requirements in university teaching may lead to a reduction in sleep hours and a decrease in sleep quality, which may trigger emotional, mental and physical health problems that contribute to the development of vocal disorders [7,53,54].

In terms of Burnout Syndrome, university professors with poor sleep quality had higher levels of emotional exhaustion and depersonalisation, as well as lower levels of self-fulfilment. The scientific literature establishes a close relationship between sleep disorders and the dimensions of emotional exhaustion and depersonalisation [16]. Studies such as Yang [19], although conducted with pre-university teachers, identify a vulnerability to emotional exhaustion in teachers with a high frequency of sleep disturbance and sleep deprivation. In addition, research confirms that burnout syndrome is associated with a lower quality of life, which in turn is related to sleep deprivation and poor sleep quality, as well as feelings of insufficient rest [55].

On the other hand, in terms of physical activity habits, teachers with poor sleep quality showed lower METS and more sedentary time per week. These results are consistent with studies that, although conducted with adolescents, associate poor sleep quality with unhealthy lifestyle habits, such as restricting and decreasing physical activity [56]. In addition, research with primary and secondary school teachers during the COVID-19 period has shown that high levels of physical activity are associated with better sleep quality, specifically that teachers with high levels of physical activity are 40% more likely to have good quality sleep [57]. A systematic review showed in healthy age groups that moderate physical activity benefits their sleep quality [58]. Similarly in reference to sedentary behaviour which is associated with insomnia and sleep disorders [19]. Although the scientific literature with samples of university professors is scarce, the studies carried out with pre-university teachers agree that lack of sleep is an important risk factor for the health and wellbeing of the teachers themselves, which in turn influences the psychosocial area, resulting in a possible transfer to the students themselves [59].

In relation to eating behaviour, teachers with poor sleep quality had higher levels of uncontrolled and emotional eating. Research such as Weiss et al., 2010 described an increased intake of snacks and calorific food, especially fats, by subjects with little sleep or poor sleep quality. Similarly, these data are confirmed by other research that concludes a more restrictive food consumption and lower quality of fibre, protein and carbohydrates by those who sleep less than 7 hours [60]. Current research with university professors speaks of inadequate eating habits in teachers who experience poor sleep quality due to excessive university workload [16,61].

One of the main strengths of this study lies in obtaining a sample of 1560 lecturers from thirteen Spanish universities, which made it possible to analyse the object of study from a broad sample of university professors at a national level. This approach provided a broad overview concerning the prevalence of sleep quality among Spanish university professors, as well as its relationship with various lifestyle habits and physical and mental health indicators. However, the research is not without limitations that need to be considered. Firstly, data collection was based on self-report questionnaires, which implies a subjective assessment by the participants. Incorporating additional clinical evaluations into this type of research has proven to be impractical, not readily available and too time-consuming to use and cost-effective. However, additional evaluations that assess more precisely the causes of changes in participants’ sleep pattern and quality would be relevant as a prospective measure, as they could provide complementary information on these changes. However, it should be noted that the measurements used have been shown to be reliable and valid in studies with similar populations. Another limitation of the study is that the use of technology before sleeping was not considered in the questionnaire. It is considered an interesting approach and could be addressed more specifically in future lines of research. Furthermore, the cross-sectional design of the study limits the ability to establish causal relationships. To address this limitation, future studies of a longitudinal nature would allow for a broader understanding and knowledge of sleep quality in the university context.

## Conclusions

The results of this study reflect a 33% rate of poor sleep quality among university professors (95% CI: 30.7% - 35.3%). Significant differences were identified based on sociodemographic factors, particularly gender, with 74.9% of men reporting good sleep quality compared to 50.5% of women. According to the regression analysis, sleep problems were associated with less improvement in vocal symptoms after rest and lower quality of life scores. Additionally, they were linked to greater vocal fatigue, physical discomfort in the voice, and higher scores in mental health disorders (stress, anxiety, and depression). University professors with poor sleep quality also showed lower levels of physical activity, increased sedentary time, and higher levels of uncontrolled and emotional eating.

Given the multitude of associations between lifestyle habits, physical and psychosocial health indicators, and sleep quality, university professors’ health promotion and prevention strategies require an interdisciplinary and multidisciplinary approach. The high demands of organisation and work development to which these professionals are subjected, accelerated work pace and great responsibility, together with the high educational demands, favour a personal and professional academic scenario of risk for the health of the teacher. Therefore, optimising rest and increasing sleep quality among university professors will promote a restoration of their physical and mental performance and contribute to greater well-being and job satisfaction. Therefore, future research is needed to investigate the physical and mental resting demands of university professors, which have an impact on their daily life and health habits. Designing health promotion and prevention programmes adjusted to the specific conditions of the university environment and being aware of this need, will benefit an optimal quality of occupational health by increasing the degree of satisfaction of the university professors and, consequently, an optimal healthy occupational and functional development of the university.
